# Mucoepidermoid Carcinoma of the Palatine Tonsil

**DOI:** 10.1155/2015/827560

**Published:** 2015-10-15

**Authors:** Lucas Novaes Teixeira, Victor Angelo Martins Montalli, Luiz Carlos Santana Teixeira, Fabrício Passador-Santos, Andresa Borges Soares, Vera Cavalcanti de Araújo

**Affiliations:** Department of Oral Pathology, São Leopoldo Mandic Institute and Research Center, Rua José Rocha Junqueira 13, Ponte Preta, 13045-755 Campinas, SP, Brazil

## Abstract

Mucoepidermoid carcinoma (MEC) is the most common primary salivary gland malignancy in both adults and children. It has a slight female predilection and usually presents as a painless, rubber-like or soft mass, which may be fixed or mobile. Histologically, MEC is comprised of a mixture of cell types including mucous, epidermoid, and intermediate cells that can be arranged in solid nests or cystic structures. In the oral cavity, it most frequently occurs at the palate or buccal mucosa. The present paper aimed to describe an unusual case of MEC arising in the palatine tonsil.

## 1. Introduction

Many different malignant neoplasms may arise from the palatine tonsils, with the most common histological type being the squamous cell carcinoma (SCC), which accounts for up to 85% of all cases [1–3]. Malignant lymphoproliferative diseases are the second most frequent malignancy of the palatine tonsil, with the diffuse large B-cell lymphoma (DLBCL) comprising approximately 30% of all lymphomas [4]. Metastatic deposits of lung [5] and gastric carcinomas [6], as well as melanoma [7], renal carcinoma [8], and adenocarcinoma of the colon [9], have also been described in the palatine tonsils.

Minor salivary gland tumors exhibit diverse histopathological features as well as a varied clinical behavior [10]. They may be derived from any of the minor salivary glands distributed throughout the oral cavity [11]. Interestingly, despite the presence of minor salivary glands in the palatine tonsils, the development of malignant salivary tumors here is unusual. Indeed, a scarce number of case reports have been documented in the scientific literature [12–14]. The present paper, therefore, aimed to report a case of MEC arising in the palatine tonsil.

## 2. Case History

**Figure 1:**
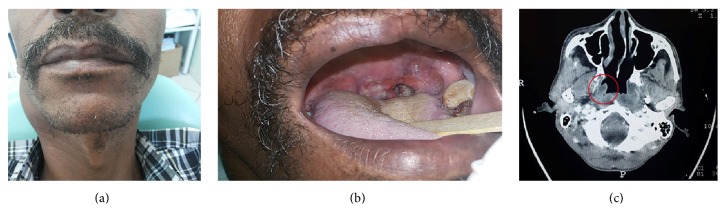
(a) The swelling on the right side of the neck, (b) ulcerated mass on the right palatine tonsil, and (c) CT image exhibiting a lobulated and ill-defined lesion (red circle).

A 47-year-old man reported experiencing dysphagia and a sore throat for 4 months. The patient was both a heavy smoker (40 cigarettes daily) and an alcoholic. His medical history was significant for Type 2 Diabetes Mellitus. The patient presented with a swelling on the right side of the neck (Figure 1(a)). Intraoral examination revealed an ulcerated mass on the right palatine tonsil (Figure 1(b)). Axial computed tomography (CT) revealed a solid lesion with lobulated and ill-defined margins (Figure 1(c)). An incisional biopsy was performed and the specimen was fixed in 10% buffered formalin.

**Figure 2:**
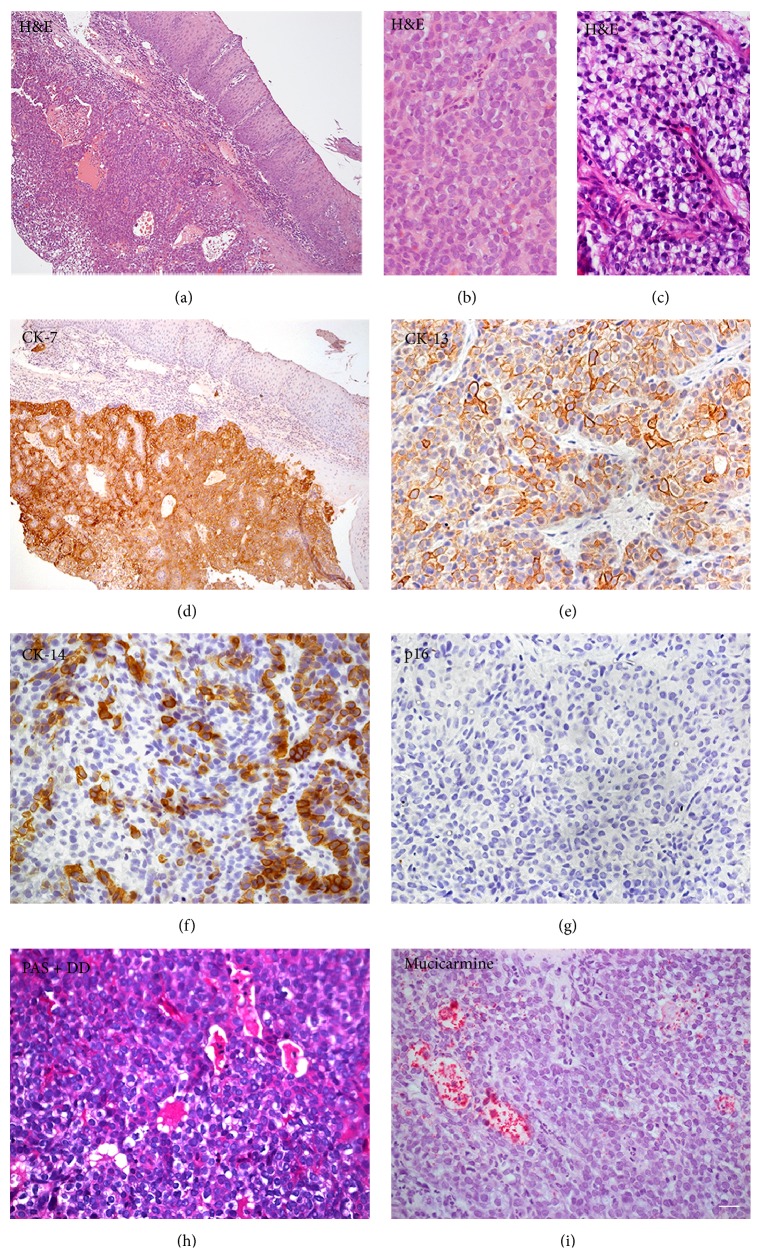
(a) H&E revealing a neoplasm of glandular epithelial origin within the lamina propria. (b) The cells showed a homogenous morphology with few cells showing atypia and mitotic figures. (c) Clear cells were observed in some areas. The neoplastic cells were positive for (d) CK-7, (e) CK-13, and (f) CK-14 and negative for (g) p16. (h) PAS with diastase digestion (i) and mucicarmine were also detected within the tumor. Scale bar: (a, c) = 80 *μ*m; (b, d, e, f, g, h) = 20 *μ*m.

Paraffin sections were prepared for light microscopy using routine procedures. The sections were stained with hematoxylin and eosin (H&E). Microscopic examination revealed a fragment of oral mucosa covered with a nonkeratinized stratified squamous epithelium. A neoplasm of glandular epithelial origin was identified in the lamina propria (Figure 2(a)). The tumor cells were arranged in sheets, exhibiting a uniform morphology with few cells showing atypia and mitotic figures (Figure 2(b)). Clear cells were observed in some areas of the tumor (Figure 2(c)).

The samples were subsequently submitted to immunohistochemistry for a subset of cytokeratins (CK), vimentin, smooth muscle actin, chromogranin, and p16, the latter indicating the presence of HPV. The source, clone, concentration, and incubation time of the primary antibodies are described in Table 1. PAS with diastase digestion (PAS + DD) and mucicarmine staining were also performed. CK-7 was positive in the neoplastic cells, while the superficial oral epithelium was negative (Figure 2(d)). CK-13 and CK-14 positivity was also observed in the neoplastic cells (Figures 2(e) and 2(f)), while vimentin, smooth muscle actin, and p16 were negative (Figure 2(g)). Some areas containing mucous were identified within the tumor, as demonstrated by positivity to PAS + DD and mucicarmine (Figures 2(h) and 2(i)). These results in combination support the diagnosis of MEC (Table 2). The patient was referred to the Hospital of the State University of Campinas (UNICAMP), where the tumor was considered inoperable. The patient underwent chemotherapy and radiotherapy but unfortunately died 6 months after diagnosis.

## 3. Discussion

MEC is one of the most common malignancies of the minor salivary glands [15–17] and is currently described as “a malignant glandular epithelial neoplasm characterized by mucous, intermediate, and epidermoid cells, with columnar, clear cell and oncocytoid features” [18]. According to histopathologic features and the relationship among its cellular components, MEC is classified as low-grade, intermediate-grade, or high-grade [19]. MEC of the minor salivary glands most frequently occurs at the palate and buccal mucosa followed by tongue, gingiva, floor of the mouth, and nasal cavity [20].

This case report describes a case of MEC affecting the right palatine tonsil. The palatine tonsils are considered part of Waldeyer's ring, whose main role is antibody synthesis [21]. Due to its position at the entrance of the oropharynx, the tonsils are the first soft tissue to encounter inhaled and ingested microorganisms. Thus, they are considered the first line of defense against exogenous aggressors [22]. A large number of tumors occur in the palatine tonsils, including SCC, the most common neoplasm of this region [3]. Histologically, SCC is characterized by marked cellular pleomorphism, abnormally large nuclei, increased nuclear to cytoplasmic ratio, and numerous typical and atypical mitotic figures, with or without necrosis [23]. In the present case, neoplastic cells exhibited a homogenous morphology, with mitoses being detected rarely.

The primary risk factors traditionally associated with the development of oral SCC development are smoking and alcohol consumption [24–26]. However, cases of SCC arising from the tonsil area may also be associated with HPV infection [27]. Indeed, approximately 51% of these carcinomas contain HPV DNA [28], with the most prevalent HPV subtype identified being HPV-16, detected in 84% HPV DNA positive tumors [28]. The chance of HPV infection associated with the development of the carcinoma described in the present case report should be discarded, since immunostaining for p16, which is indicative of the presence of HPV, was negative.

Adenocarcinomas account for less than 1% of all malignancies of the palatine tonsils [3], with only a few studies having described the presence of such tumors at this site. The most frequent histological subtypes are represented by adenoid cystic carcinoma [13, 14] and polymorphous low-grade adenocarcinoma [12]. Regarding MEC, only two cases arising in the palatine tonsils have been described in the literature [29, 30].

Approximately 70% of all cases of MEC are comprised by low-grade and intermediate-grade tumors [31, 32]. Thus, routine diagnosis of MEC is generally based on conventional H&E staining looking to identify the presence of cystic structures and mucous cells [20]. High-grade MEC, however, can resemble SCC, as observed in the present case report. In this context, immunohistochemical analysis represents a useful diagnostic tool for salivary gland tumors [33]. The use of this technique to improve understanding of salivary gland tumors was initiated in the 1980s with studies of intermediate keratin filaments, vimentin, and desmin [34–39]. Keratins are a group of intermediate filaments restricted to the epithelium, which are preserved in both malignant transformation and metastasis [40, 41]. During the development of normal human minor salivary glands, one may observe the expression of different CK subtypes [42]. CK-7 was the first CK identified in salivary glands, with its expression remaining in adult salivary glands [38, 42, 43]. In the present case report, immunohistochemistry revealed the presence of large areas showing positivity to CK-7, confirming its glandular origin. Interestingly, Regauer et al. detected a subset of CK-7 positive carcinomas in the Waldeyer's ring area [44]. They suggested that these neoplasms are better classified as basaloid SCC. Although CK-7 was reported in this case report, the morphologic characteristics of basaloid SCC were not encountered, namely, lobules of basaloid cells with central areas of comedonecrosis [45].

Immunohistochemistry for the present case indicated that, besides CK-7, the neoplastic cells were also positive for CK-13 and CK-14, as described previously [33, 46]. Some authors have suggested that positivity for CK-14 may be a distinctive marker in the diagnosis of oral SCC [47, 48]. However, one should note that this CK can also be detected in MEC [33, 46]. Indeed, the basal cells of the epithelium of the oral mucosa, as well as the basal cells of the excretory duct in normal salivary tissues, are positive for CK-14 [47–49]. Thus, positive immunostaining for CK-14 is most likely related to the excretory duct origin of MEC [50]. Moreover, the neoplastic cells exhibited CK-7 positivity, which excludes the chance of SCC. The expression of CK-13 may be detected in MEC, since luminal cells of excretory ducts are positive for this CK, which may also be used as a marker in the differential diagnosis of other salivary gland tumors [33]. In addition to the CKs, staining with PAS + DD and mucicarmine can also be useful markers to discriminate SCC from MEC [51]. Positive areas of PAS and mucicarmine detected in some areas of the tumor corroborated the diagnosis of MEC described in the present case report.

The standard approach for the treatment of MEC is total excision of the tumor [52]. However, minor salivary gland carcinomas of the oropharynx are often challenging to manage [53], with radical tonsillectomy and ipsilateral neck dissection having been used as a treatment option [30, 54]. In the present case, due to the advanced stage of the tumor at presentation, the treatment of choice was chemo- and radiotherapy. Some studies have advised the use of radiotherapy for both high-grade MEC and patients with unclear surgical margins [55–57]. On the other hand, the data regarding chemotherapy in MEC is scarce, with this treatment generally being adopted in palliative management of inoperable tumors [58]. Despite the use of these two modalities in the treatment of the present case, the patient unfortunately died.

In conclusion, this case report highlights the possibility of salivary gland tumor affecting the oropharynx. Thus, during the differential diagnosis of a tumor mass at this specific site, whether asymptomatic or not, salivary gland tumors should not be ignored, since early diagnosis and appropriate management are a determining factor in the prognosis of the patient.

## Figures and Tables

**Table 1:** Specifications of the primary antibodies.

Primary antibody	Source	Dilution	Retrieval	Incubation time
CK-7	Dako	1 : 100	Water bath 95°C (citric acid): 30 min	60 min
CK-13	Dako	1 : 100	Water bath 95°C (citric acid): 30 min	60 min
CK-14	NeoMarkers	1 : 1200	Water bath 95°C (citric acid): 30 min	60 min
Vimentin	Dako	1 : 300	Water bath 95°C (citric acid): 30 min	60 min
Smooth muscle actin	Dako	1 : 100	Water bath 95°C (citric acid): 30 min	60 min
Chromogranin	Abcam	1 : 500	Water bath 95°C (citric acid): 30 min	60 min
p16	CINtec Histology	1 : 250	Water bath 95°C (citric acid): 30 min	60 min

**Table 2:** Immunohistochemistry results.

Primary antibody	Result
CK-7	+
CK-13	+
CK-14	+
Vimentin	−
Smooth muscle actin	−
Chromogranin	−
p16	−
